# Case Report: Pneumonia in a Patient With Combined Variable Immunodeficiency: COVID-19 or Pneumocystis Pneumonia?

**DOI:** 10.3389/fmed.2022.814300

**Published:** 2022-02-23

**Authors:** Shabnam Tehrani, Shadi Ziaie, Alireza Kashefizadeh, Mahta Fadaei, Hanieh Najafiarab, Amirreza Keyvanfar

**Affiliations:** ^1^Infectious Diseases and Tropical Medicine Research Center, Shahid Beheshti University of Medical Sciences, Tehran, Iran; ^2^Department of Clinical Pharmacy, School of Pharmacy, Shahid Beheshti University of Medical Sciences, Tehran, Iran; ^3^Shahid Dr. Labbafinejad Hospital, Shahid Beheshti University of Medical Sciences, Tehran, Iran; ^4^School of Medicine, Shahid Beheshti University of Medical Sciences, Tehran, Iran; ^5^Preventative Gynecology Research Center, Shahid Beheshti University of Medical Sciences, Tehran, Iran

**Keywords:** common variable immunodeficiency (CVID), COVID-19, SARS-CoV-2, Pneumocystis pneumonia, case report

## Abstract

Combined variable immunodeficiency (CVID) is a primary immunodeficiency, characterized by impairment in immune system function. These patients are susceptible to opportunistic infections, which may mimic COVID-19 manifestations. Also, misdiagnosis or delayed diagnosis of opportunistic infections can lead to perilous consequences. We report a 28-year-old woman with a history of combined variable immunodeficiency disorder (CVID) and ulcerative colitis (UC) complained of fever, cough, and dyspnea. According to the clinical and radiological manifestations and the COVID-19 epidemic, she was admitted with a primary diagnosis of COVID-19 pneumonia. After a week, the patient did not respond to treatment, so she underwent bronchoscopy. Using polymerase chain reaction (PCR) methodology, we detected DNA of *Pneumocystis jirovecii*, the causative agent of a life-threatening pneumonia (PCP), in respiratory specimens. The patient was hypersensitive to common PCP treatments, so she was treated with high-dose clindamycin. However, the patient's clinical condition aggravated. Besides, we found evidence of pneumothorax, pneumomediastinum, and pneumopericardium in chest CT scan. We inserted a catheter for the patient to evacuate the air inside the mediastinum. Also, we added caspofungin to the treatment. The patient eventually recovered and was discharged from the hospital about a week later. Thus, during the COVID-19 epidemic, in febrile patients with respiratory symptoms, physicians should not think only of COVID-19. They must consider opportunistic infections such as PCP, especially in immunocompromised patients.

## Introduction

Combined variable immunodeficiency disorder (CVID) is the most common primary immunodeficiency worldwide (1). These patients have a broad spectrum of B and T cell dysfunctions, so their immunological responses are impaired, and they are prone to a variety of infections (2). Severe acute respiratory syndrome-coronavirus-2 (SARS-CoV-2), like intact individuals, can infect patients with CVID. Few studies have reported that CVID patients are not different from other people considering mortality and severity (3). Due to impaired immune responses, these patients are less likely to have a cytokine storm and severe complications of COVID-19. Instead, they are prone to opportunistic infections, which can mimic COVID-19 manifestations (4). For this reason, among immunocompromised patients, it is important to distinguish accurately COVID-19 from the differential diagnoses such as *P.jirovecii* and Cytomegalovirus (CMV) infections. Misdiagnosis and delayed diagnosis of opportunistic infections can lead to perilous consequences, even death (5).

## Case Description

A 28-year-old woman complaining of fever, cough, and dyspnea was admitted in June 2021 to Labbafinejad hospital, Tehran, Iran. Symptoms began 14 days earlier and gradually worsened. The most important point in her medical history was the diagnosis of CVID 6 years ago, under treatment with Intravenous immune globulin (IVIG) monthly. She also was a known case of ulcerative colitis and was being treated with azathioprine, mesalazine, and prednisolone. Additionally, 1 week before this visit, the patient was diagnosed with CMV viremia, was treated with valganciclovir. At the time of admission, her vital signs were as follows: respiratory rate = 30 /min, pulse rate = 116 /min, blood pressure = 100/65, temperature = 38°C, and Saturation of peripheral oxygen (SpO_2_) on room air = 90%.

The patient underwent laboratory tests and imaging. The Reverse transcription polymerase chain reaction (RT-PCR) result from nasopharyngeal secretions was negative for SARS-CoV-2. RT-PCR was used for amplifying RNA-dependent RNA polymerase (RdRp), envelope (E), and nucleocapside (N) genes. Table 1 presents the laboratory findings of the patient. The spiral chest Computed tomography (CT) scan showed bilateral ground-glass opacities (compatible with COVID-19 pneumonia) and multiple nodules (consistent with CMV pneumonia or fungal infections) (6, 7). Thus, to cover all the differential diagnoses mentioned above, we prescribed the following drugs:

remdesivir (200 mg daily for first day and 100 mg daily for the next 4 days),
dexamethasone (8 mg daily for 5 days),
voriconazole (6 mg/kg every 12 h for first day and 4 mg/kg every 12 h for the next 7 days),

and replacing injectable ganciclovir (5 mg/kg every 12 h for first day and 5 mg/kg daily for the next 13 days) instead of valganciclovir. Also, throughout the hospitalization, the patient received oxygen with a simple mask (6–10 L/min).

**Table 1:** Laboratory tests of the patients during hospitalization.

**Variables**	**Normal values**	**1st day**	**12th day**	**19th day (discharge)**
CRP (mg/dL)	<10, Negative	11	14	1.2
LDH (U/L)	<250	582	680	362
WBC (10^3^/μL)	4–10	8.3	7.1	5.2
Neutrophil (%)	47–76	85	95	90
Lymphocyte (%)	25–45	10	5	7
Hb (mg/dL)	12–16	16.4	13.0	12.1
PLT (10^3^/μL)	150–450	188	277	255
VBG				
PH	7.30–7.40	7.29	7.38	7.30
PCO_2_ (mmHg)	40–50	41	28	35
HCO_2_ (mmol/L)	22–28	19.7	18.5	20.3

*CRP, c-reactive protein; LDH, lactate dehydrogenase; WBC, white blood cell; Hb, hemoglobin; PLT, platelet; VBG, venous blood gas*.

On the fifth day of hospitalization, dyspnea and tachypnea improved relatively; however, she was still coughing with no change in her oxygen saturation. Also, no fever was detected since the third day of hospitalization. On the eighth day of hospitalization, the patient did not respond to treatment as expected. In addition, the patient complained of chest pain and had subcutaneous chest emphysema on examination. Therefore, we performed more diagnostic measures. Repeated chest CT scan showed that the ground-glass opacities and multiple nodules were reduced. In addition, pneumomediastinum, limited pneumothorax, and subcutaneous emphysema were reported. The patient then underwent bronchoscopy. Bronchoalveolar lavage was negative for *Aspergillus* and galactomannan (fungal infections ruled out) and SARS-CoV-2 PCR (by targeting RdRp, E, and N genes). But it turned out to be positive for Pneumocystis pneumonia (PCP) genome by PCR. PCR method was used for amplifying mitochondrial large subunit rRNA (mt-LSU-rRNA). So, the patient was discontinued antifungal medication. We first prescribed co-trimoxazole to treat PCP, but the patient had severe hypersensitivity. After discontinuation, a combination of clindamycin and primaquine was administered. The patient was also hypersensitive to primaquine. So, considering pharmacological consult, treatment with high-dose clindamycin (600 mg every 8 h for 7 days) was continued. Additionally, based on surgical consult, high-pressure oxygen was prescribed to treat pneumothorax and pneumomediastinum.

**Figure 1:**
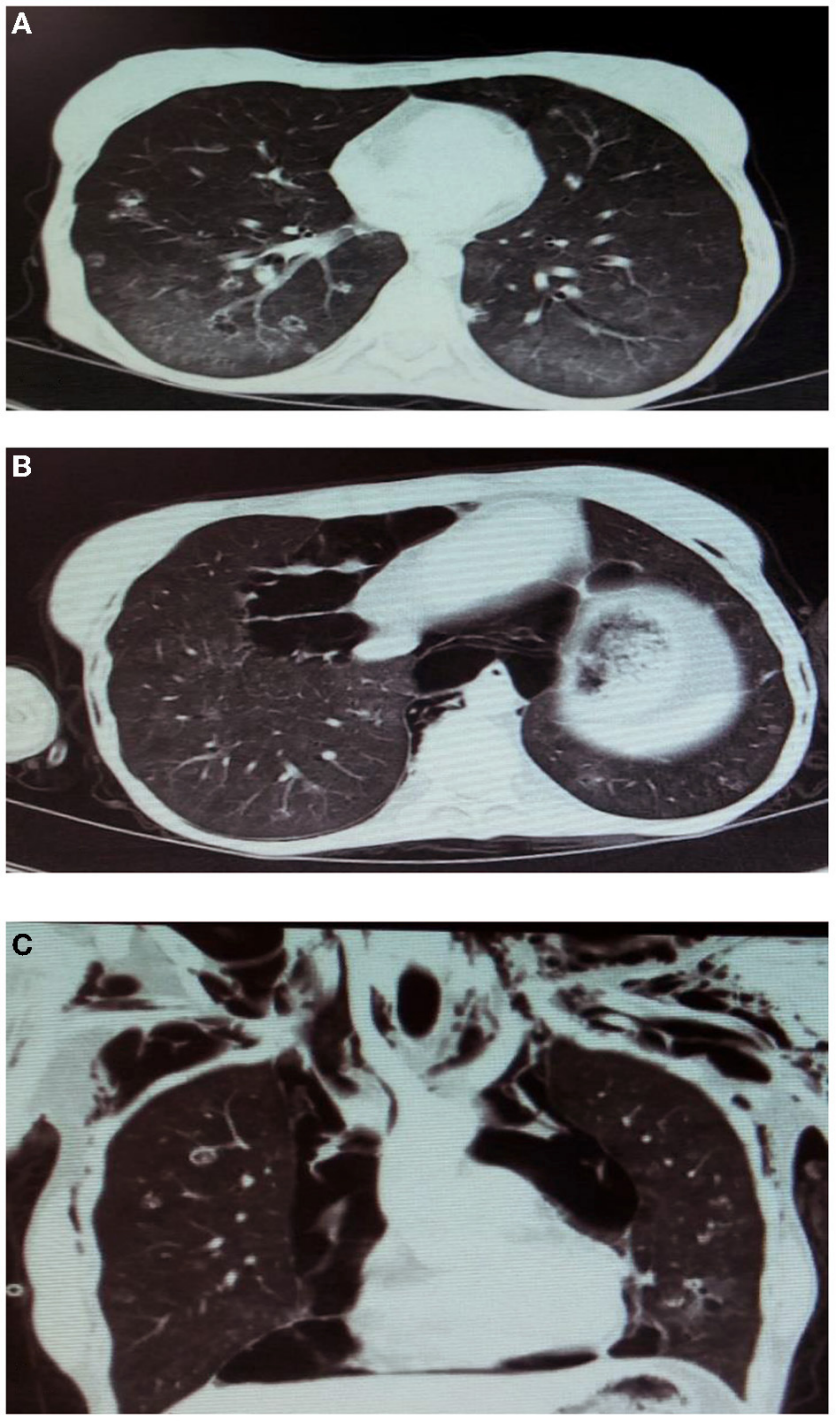
The spiral chest CT scan of the patient. **(A)** 1st day of hospitalization; bilateral ground-glass opacities and multiple nodules. **(B,C)** 12th day of hospitalization,pneumothorax, pneumomediastinum, extensive subcutaneous emphysema, and pneumopericardium.

On the 12th day of hospitalization, despite no drop in oxygen saturation, the cough and dyspnea intensified. Further CT scan showed enlargement of the pneumothorax and pneumomediastinum, extensive subcutaneous emphysema, and pneumopericardium (Figure 1). Due to the pneumopericardium, a catheter was inserted for 24 h to expel the air. Also, we added caspofungin (50 mg daily for 7 days) to the previous anti-PCP treatment.

On the 19th day of hospitalization, the patient was treated with anti-PCP and anti-CMV regimens. The patient's clinical symptoms improved, vital signs on the examination were stable. The last CT scan showed no pneumothorax, pneumopericardium, or pneumomediastinum, and the lung parenchyma was completely normal. Eventually, we discharged the patient in good general condition.

## Discussion

In patients with CVID, humoral immunity is usually impaired, while cellular immunity is somewhat efficient. Hypogammaglobulinemia is common in CVID patients, making them more susceptible to recurrent respiratory and gastrointestinal infections. They become more infected with bacterial pathogens due to impaired humoral immunity. In contrast, fungal and viral pathogens are less common in them, as they are more likely to infect people with cellular immunodeficiency (8).

The case presented also had a history of UC, and the drugs mentioned for its treatment cause impairment of cellular immunity. As a result, opportunistic respiratory infections caused by CMV, fungi, and tuberculosis should be considered differential diagnoses of the above patient (9, 10).

There are two explanations for the clinical course of the reported patient that are difficult for the clinicians to distinguish. First, the patient may have been a case of COVID-19 from the beginning, followed by PCP superinfection. Considering the COVID-19 epidemic, as well as the mentioned clinical characteristics and the ground-glass opacities found in the CT scan, the patient was diagnosed with COVID-19 pneumonia. However, due to the inappropriate response to treatment and the background of immunodeficiency, we performed bronchoscopy. Then PCP was diagnosed using PCR. This patient with a history of under-treatment CVID and UC already had a CMV viremia that can aggravate immunodeficiency. Besides, COVID-19 and the medications used to treat it interfere with the immune system. All of these risk factors make the patient susceptible to opportunistic infections such as PCP (11).

Second, the patient could have PCP from the start, misdiagnosed as COVID-19. The clinical manifestations of pneumonia caused by SARS-CoV-2 and *P.jirovecii* are similar (6). Both of them show bilateral ground-glass opacities in lung CT scan. As a result, they may be misdiagnosed instead (12). In other words, from the beginning, our patient was a case of pneumonia caused by CMV and *P.jirovecii*, which improved after anti-CMV treatment. But abruptly, the pneumonia caused by *P.jirovecii* progressed, exacerbating the clinical symptoms and causing pneumothorax and pneumomediastinum.

In previous studies, up to 4% of non-HIV patients with PCP developed spontaneous pneumothorax. Pneumothorax in these patients is related to the following explanations: Neutrophilic lung inflammation caused by PCP may lead to paranchymal pneumatocele formation. Besides, subpleural necrosis may lead to bronchopleural fistula. Also, pneumomediastinum likely results from pneumatocele rupture, and air leakage from ruptured pneumatoceles into the hilum and mediastinum (13).

After the 12th day of hospitalization, the patient recovered, which can be attributed to the following factors: There are three options to treat PCP: co-trimoxazole, clindamycin-primaquine, and pentamidine (14). Interestingly, the patient showed hypersensitivity to the first two, and pentamidine was not available. Therefore, we prescribed high-dose clindamycin with caspofungin. Caspofungin inhibits the enzyme β (1,3)-D-glucan synthase in the cell wall improving clearance of *P. jirovecii* in cases of infection by this opportunistic fungus (15–17). And as we saw, by adding it to the medication, we had an appropriate response (symptoms improvement and reduction in serum levels of C-reactive protein and Lactate dehydrogenase). Also, the patient, meanwhile, received her monthly IVIG, which helps control pneumonia (18).

## Conclusion

In conclusion, during the COVID-19 epidemic, in febrile patients with respiratory symptoms, physicians should not think only of COVID-19. Physicians should hypothesize that they are facing opportunistic infections such as PCP, especially in immunocompromised patients.

## Data Availability Statement

The original contributions presented in the study are included in the article/supplementary material, further inquiries can be directed to the corresponding author.

## Ethics Statement

Written informed consent was obtained from the patient for the publication of any potentially identifiable images or data included in this article.

## Author Contributions

ST and AKa were the treating physicians of the patient. MF and HN collected all the medical history of the patient. AKe wrote the core of this article and conceptualization. SZ gave consultative opinion on clinical pharmacology. All authors have read and endorsed the final draft.

## Conflict of Interest

The authors declare that the research was conducted in the absence of any commercial or financial relationships that could be construed as a potential conflict of interest.

## Publisher's Note

All claims expressed in this article are solely those of the authors and do not necessarily represent those of their affiliated organizations, or those of the publisher, the editors and the reviewers. Any product that may be evaluated in this article, or claim that may be made by its manufacturer, is not guaranteed or endorsed by the publisher.

## References

[1] Weifenbach N Jung A Lötters S. COVID-19 infection in CVID patients: What we know so far. Immun Inflamm Dis. (2021) 9:632–4. 10.1002/iid3.450 33979068 PMC8239877

[2] Ameratunga R Longhurst H Steele R Lehnert K Leung E Brooks AES. Common variable immunodeficiency disorders, T-cell responses to SARS-CoV-2 vaccines, and the risk of chronic COVID-19. J Allergy Clin Immunol Pract. (2021) 9:3575–83. 10.1016/j.jaip.2021.06.019 34182162 PMC8230758

[3] Marcus N Frizinsky S Hagin D Ovadia A Hanna S Farkash M. Minor clinical impact of COVID-19 Pandemic on patients with primary immunodeficiency in Israel. Front Immunol. (2021) 11:3505. 10.3389/fimmu.2020.614086 33519822 PMC7840610

[4] Liu BM Hill HR. Role of host immune and inflammatory responses in COVID-19 cases with underlying primary immunodeficiency: a review. J Interferon Cytokine Res. (2020) 40:549–54. 10.1089/jir.2020.0210 33337932 PMC7757688

[5] Coleman JJ Manavi K Marson EJ Botkai AH Sapey E. COVID-19: to be or not to be; that is the diagnostic question. Postgrad Med J. (2020) 96:392–8. 10.1136/postgradmedj-2020-137979 32522844 PMC7306267

[6] Szydłowicz M Matos O. Pneumocystis pneumonia in the COVID-19 pandemic era: similarities and challenges. Trends Parasitol. (2021) 37:859–62. 10.1016/j.pt.2021.07.010 34364804 PMC8335554

[7] Merchant EA Flint K Barouch DH Blair BM. Co-infection with coronavirus disease 2019, previously undiagnosed human immunodeficiency virus, Pneumocystis jirovecii pneumonia and cytomegalovirus pneumonitis, with possible immune reconstitution inflammatory syndrome. IDCases. (2021) 24:e01153. 10.1016/j.idcr.2021.e01153 33977081 PMC8103711

[8] Zainaldain H Rizvi FS Rafiemanesh H Alizadeh M Jamee M Mohammadi S. Infectious complications reporting in common variable immunodeficiency: a systematic review and meta-analysis. Oman Med J. (2020) 35:e157. 10.5001/omj.2020.64 32802416 PMC7417520

[9] Beaugerie L Rahier JF Kirchgesner J. Predicting, preventing, and managing treatment-related complications in patients with inflammatory bowel diseases. Clin Gastroenterol Hepatol. 2020;18(6):1324–35.e2. 10.1016/j.cgh.2020.02.009 32059920

[10] Watanabe Y Hayashi K Terai S A. Rare case of ulcerative colitis with severe pneumocystis jirovecii pneumonia and cytomegalovirus colitis: a case report and literature review. Internal Med (Tokyo, Japan). (2021). 10.2169/internalmedicine.7953-21 34373380 PMC8866792

[11] Abdoli A Falahi S Kenarkoohi A. COVID-19-associated opportunistic infections: a snapshot on the current reports. Clin Exp Med. (2021) 1–20. 10.1007/s10238-021-00751-7 34424451 PMC8381864

[12] Schüßler M Müller F Rauschning D. [Not all cases of groundglas opacity are COVID-19 - Pneumocystis-jirovecii-pneumonia as a differential diagnosis]. Deutsche medizinische Wochenschrift. (1946) 146:603–7. 10.1055/a-1391-4403 33931838

[13] Yee D Fu D Hui C Dharmadhikari N Carino G. A rare case of 4 ps: bilateral pneumothoraces and pneumomediastinum in pneumocystis pneumonia. Rhode Island Med J. (2013) 103:52–4. 32481782

[14] Weyant RB Kabbani D Doucette K Lau C Cervera C. Pneumocystis jirovecii: a review with a focus on prevention and treatment. Expert Opin Pharmacother. (2021) 22:1579–92. 10.1080/14656566.2021.1915989 33870843

[15] Farhadi Z Farhadi T Hashemian SM. Virtual screening for potential inhibitors of β(1,3)-D-glucan synthase as drug candidates against fungal cell wall. J Drug Assess. (2020) 9:52–9. 10.1080/21556660.2020.1734010 32284908 PMC7144292

[16] Abolghasemi S Sharif-Kashani B Naghashzadeh F Marjani M Moniri A Doroudinia A. Caspofungin as salvage therapy for pneumocystis pneumonia in a heart transplant recipient. Tanaffos. (2018) 17:203–6. 30915138 PMC6428377

[17] Lobo ML Esteves F de Sousa B Cardoso F Cushion MT Antunes F. Therapeutic potential of caspofungin combined with trimethoprim-sulfamethoxazole for pneumocystis pneumonia: a pilot study in mice. PLoS ONE. (2013) 8:e70619. 10.1371/journal.pone.0070619 23940606 PMC3734247

[18] Melo KM Alves LM Valente CFC Tavares FS. One-year intravenous immunoglobulin replacement therapy: efficacy in reducing hospital admissions in pediatric patients with inborn errors of immunity. J Pediatr. (2021). 10.1016/j.jped.2021.05.011. [Epub ahead of print]. 34273274 PMC9432171

